# 70 Years of DON and Beyond: Glutaminase Inhibition as a Synergistic Strategy in Cancer Combination Therapy

**DOI:** 10.3390/pharmaceutics18070850

**Published:** 2026-07-13

**Authors:** José A. Campos-Sandoval, Juan De los Santos-Jiménez, Javier Márquez, José M. Matés

**Affiliations:** 1 Canceromics Lab, Departamento de Biología Molecular y Bioquímica, Universidad de Málaga, 29071 Málaga, Spain; jacs@uma.es (J.A.C.-S.); jsj@uma.es (J.D.l.S.-J.); marquez@uma.es (J.M.); 2 Instituto de Investigación Biomédica de Málaga (IBIMA-Plataforma BIONAND), Universidad de Málaga, 29071 Málaga, Spain; 3 Department of Biochemistry and Molecular Biology I, University of Granada, 18071 Granada, Spain; 4 Centre for Genomics and Oncological Research Pfizer (GENYO), University of Granada, Andalusian Regional Government, 18071 Granada, Spain; 5 Instituto de Investigación Biosanitaria de Granada (ibs.GRANADA), 18012 Granada, Spain

**Keywords:** DON, BPTES, CB-839, combination therapy, DRP-104, glutaminase, synergistic effects

## Abstract

Personalized oncology seeks to selectively block specific dysregulated pathways to arrest cancer development. Increased glutamine metabolism is a hallmark of cancer, and 6-diazo-5-oxo-L-norleucine (DON), a structural analog of L-glutamine, was the first compound used to target the exacerbated nitrogen metabolism observed in cancer cells. However, its clinical application was limited by unacceptable toxicity. With the same goal of blocking glutamine metabolism, several specific glutaminase inhibitors have been characterized in recent decades, showing promising antitumor activity. Nevertheless, this strategy frequently induces adaptive metabolic resistance that must be counteracted. In this context, glutaminase has become a key target in combination therapies for several tumor types aimed at restricting anabolic adaptation when single metabolic therapy fails, emerging as a possible synergistic therapeutic intervention. Consequently, combination therapies that include glutaminase inhibition alongside additional agents to counteract the metabolic plasticity of cancer have emerged as a promising approach in personalized antitumor pharmacology. This review provides a historical-to-translational overview of glutamine-targeted therapies, with particular emphasis on glutaminase inhibitors, including compound 968, BPTES, CB-839, and next-generation inhibitors, as well as DON-derived prodrugs. We discuss their mechanisms of action and their integration with chemotherapy, targeted therapies, radiotherapy, and immunotherapy, highlighting how glutamine metabolism targeting influences tumor metabolic adaptation, redox homeostasis, therapy resistance, and tumor–immune interactions. Finally, we examine current clinical developments, emerging therapeutic combinations, and the challenges that must be addressed for the incorporation of glutamine metabolism targeting into precision oncology.

## 1. Introduction

After glucose, glutamine (Gln) is the second most avidly consumed molecule by cancer cells, with most tumors showing increased Gln metabolism. Thus, approaches aimed at blocking nitrogen metabolism arose as essential antitumor strategies against cancerous growth and proliferation [1,2]. In 1956, the discovery and biological studies of 6-diazo-5-oxo-L-norleucine (DON) were published for the very first time [3], and shortly after its isolation and characterization were also reported [4], all of which proved DON to be a new useful drug in anticancer therapy. In 1957, the first clinical assay using DON as monotherapy for patients with serious malignancies (mainly lung and breast cancers) was published, but very significant side effects were observed, including ulceration of the tongue, mouth, and lips, diarrhea, nausea, and vomiting [5]. DON was later identified as a structural analog of Gln and its chemotherapeutic usefulness was fully established in the 1970s [6], with later experiments proving the synergistic effects of combining Gln depletion strategies and DON (and another Gln analog, acivicin, chemically (2S)-2-amino-2-[(5S)-3-chloro-4,5-dihydro-1,2-oxazol-5-yl]acetic acid) as powerful molecules inhibiting cancer growth [7]. In the 1980s, several studies reported promising findings in both in vitro and in vivo models, but subsequent clinical trials produced unpromising results due to low specificity (inhibition of enzymes whose substrate is Gln, amino acid transporters, and transglutaminase), likely leading to the observed generalized toxicity [8]. Nowadays, less toxic agents have been described and characterized as valuable drugs to inhibit glutaminase (GA) [9,10,11], including natural products that have shown interactions by molecular dynamics simulations but still need to be tested in more in-depth studies [12], as well as derivatives of a natural molecule, withangulatin A, that have already shown antitumoral properties in vitro and in mouse xenografts in the highly Gln-dependent triple-negative breast cancer (TNBC) subtype [13]. However, this review will be focused on fully characterized GA inhibitors, including compound 968, BPTES, and CB-839 (telaglenastat), as well as novel prodrugs that release the inhibitor at the tumor site with maximum efficacy while minimizing toxicity. On the other hand, we emphasize the importance of GA in the metabolic network of tumor cells and its crosstalk with other key molecular pathways, as well as its essential role in the regulatory signals that govern cancer metabolism and modulate the energetic and biosynthetic pathways that need to be targeted in anticancer pharmacological therapy [1,9].

**Figure 1:**
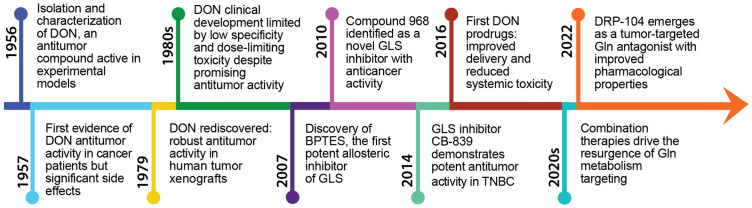
Timeline of milestones in the history of DON and glutaminase inhibitors with relevance to pharmaceutics and translation to clinic. Fundamental scientific findings are highlighted. Milestones are based on Coffey et al., 1956 [3], Dion et al., 1956 [4], Magill et al., 1957 [5], Anthony et al., 2024 [11], Ovejera et al., 1979 [14], Lynch et al., 1982 [15], Earhart et al., 1990 [16], Robinson et al., 2007 [17], Wang et al., 2010 [18], Gross et al., 2014 [19], Rais et al., 2016 [20], Wang et al., 2020 [21], Cyriac and Lee, 2024 [22], Yokoyama et al., 2022 [23], Rais et al., 2022 [24]. Gln, L-glutamine. GLS, glutaminase. TNBC, triple-negative breast cancer.

In this review, we focus on recent findings and examine how the historical evolution of DON and GA inhibitors can inform current therapeutic development in oncology. Although several comprehensive reviews have described the role of Gln metabolism in cancer, the biological relevance of glutaminolysis, and the development of GA inhibitors, these studies have primarily focused on metabolic reprogramming, the molecular regulation of Gln utilization, or the pharmacological properties of individual inhibitors. Building upon these previous contributions, the present review provides a complementary historical-to-translational perspective, integrating the evolution of Gln-targeted therapies from the discovery of DON to the development of selective GA inhibitors and next-generation DON-derived prodrugs. Particular emphasis is placed on the emergence of GA inhibition as a therapeutic strategy in combination with other anticancer approaches. We discuss how targeting Gln metabolism can enhance the efficacy of chemotherapy, targeted therapies, radiotherapy, and immunotherapy by affecting tumor metabolic plasticity, redox homeostasis, therapy resistance mechanisms, and tumor–immune interactions. By integrating historical perspectives (Figure 1), mechanistic insights, preclinical evidence, and emerging clinical data, this review highlights the opportunities and challenges associated with incorporating Gln-targeted therapies into modern precision oncology strategies.

## 2. Glutaminase Is a Metabolic Target Against Cancer

Cancer metabolism might be described as a large city underground map, where, when one pathway is blocked, another can be activated to meet the energetic and biosynthetic requirements of cancer cells. Metabolic plasticity is a hallmark of tumors and contributes to the emergence of resistance mechanisms that limit therapeutic efficacy. However, although broad, metabolic plasticity is limited, and blocking several related pathways may circumvent resistance, aiming to synergistically arrest cancer development [25,26]. In this context, GA has been fully characterized as a key target in the metabolic therapy of cancer [27,28,29]. GA is the enzyme responsible for catalyzing the conversion of Gln to glutamate, releasing the amide group from Gln as free ammonia, and represents the first step in glutaminolysis [10]. Glutaminolysis is a commonly upregulated pathway in cancer cells, as it can provide both energy (via generation of α-ketoglutarate and incorporation into the tricarboxylic acid cycle) and biosynthetic precursors for the synthesis of proteins, lipids, and nucleic acids, positioning Gln as a crucial metabolite boosting survival, accelerated growth, and proliferation [30]. Given that the reaction catalyzed by GA is the gate to downstream glutaminolytic metabolism, intense focus has been placed on inhibiting GA as an alternative or parallel target for potential cancer treatment regimens, as this strategy might help tackle the heterogeneity among cancer cells [9,31,32,33]. Several human GA proteins have been identified, encoded by two paralogous genes, GLS and GLS2 [9], each of which gives rise to two different isoforms [21]. The transcripts known as KGA (kidney-type glutaminase) and GAC (glutaminase C) arise by alternative splicing of the GLS gene, being collectively named GLS; whereas two GLS2 transcripts have also been identified: the canonical long transcript termed GAB (glutaminase B) and the short transcript LGA (liver-type glutaminase), which was originally identified in rat liver. Both GAB and LGA can also be indistinctly referred to as GLS2 isozymes [30].

GLS is consistently associated with Gln addiction in tumors and has well-established oncogenic properties; however, the role for GLS2 appears to be more complex, as it has been described as a context-dependent tumor suppressor factor [9,27,28,29]. The heterogeneity in the expression of key metabolic genes—like GLS and GLS2—suggests that different tumors might have differential requirements for glutaminolysis [34]. Accordingly, increased Gln catabolism in mouse liver tumors was associated with decreased levels of Gln synthetase (GS) and a shift from GLS2 to GLS. In sharp contrast, MYC-induced lung tumors display increased expression of both GS and GLS and accumulate Gln. These and other findings make it clear that the tumor metabolic profiles depend on both the genotype and tissue of origin, with critical implications for the design of therapies targeting tumor metabolism [35]. Anyhow, specific GLS inhibition has triggered crucial effects in the therapy of different cancers, as will be reviewed in this article. Importantly, GLS not only regulates the availability of Gln to the tumor but also acts as a tumor supportive protein in many types of cancer, as it has an important impact in pro-tumorigenic signaling [36]. The metabolic shift toward glutaminolysis—and the implications that it has for cancer progression— is driven by oncogene c-Myc-mediated GLS overexpression in many cancers [37,38,39]. Notably, c-Myc coordinates key metabolic pathways, including glycolysis and glutaminolysis. Interestingly, in prostate cancer cells, the crosstalk between these two pathways is mediated by GLS. Specifically, c-Myc-induced GLS indirectly repressed thioredoxin-interacting protein (TXNIP), thereby increasing glucose uptake and glycolysis. Inhibition of GLS restored TXNIP expression and reduced glucose uptake in PC3 cells [40]. Metabolic reprogramming in cancer cells relies on the overexpression of key enzymes that upregulate key pathways such as glycolysis, glutaminolysis, and fatty acid synthesis [1]. Combined inhibition of three enzymes essential in these pathways—hexokinase-2, GLS and fatty acid synthase— with lonidamine, DON, and orlistat, respectively, results in a significant reduction in cell viability in the human colon cancer SW480 cell line, as well as in vivo in mice. This approach shows good tolerance, overcomes resistance mechanisms, and produces synergistic effects [41]. This example illustrates a widely adopted strategy in cancer research: combination therapy.

**Figure 2:**
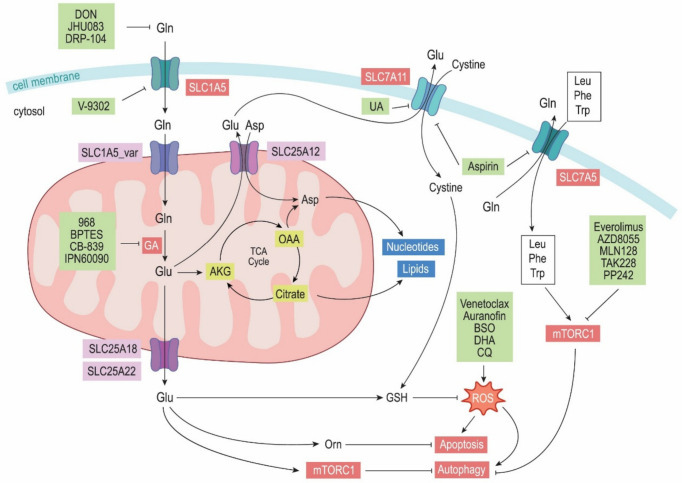
Main pathways of Gln transport and metabolism in the cytosol and mitochondria of cancer cells. Druggable targets for combination therapy are indicated in red, and corresponding drugs are shown in green. Pointed arrows indicate activation and blunt-end lines denote inhibition. AKG, alpha-ketoglutarate. Asp, aspartate. BSO, L-buthionine sulfoximine. CQ, chloroquine. DHA, dihydroartemisinin. GA, glutaminase. Gln, glutamine. Glu, glutamate. GSH, glutathione. Leu, leucine. mTORC1, mammalian target of rapamycin complex 1. OAA, oxaloacetate. Orn, ornithine. Phe, phenylalanine. ROS, reactive oxygen species. TCA, tricarboxylic acid. Trp, tryptophan. UA, ursodeoxycholic acid.

In Figure 2, we depict the main circuits involved in Gln homeostasis in cancer cells, along with major targets susceptible to pharmacological intervention within a multi-target chemotherapeutic approach. On the one hand, glutaminolysis is inhibited with a specific inhibitor, among which compound 968, BPTES, and CB-839 have shown the best results (Section 3, Section 4 and Section 5). In addition, DON prodrugs and next-generation compounds have emerged as key tools for combination therapies (Section 6, Section 7 and Section 8). On the other hand, other key proteins, metabolic pathways, cellular processes, and signaling pathways must also be targeted to effectively constrain tumor growth [10]. In the following sections, we describe GA inhibitors to better understand their effects in monotherapy and, especially, in combination therapies, looking for strategies that not only enhance the antitumor activity of the other drug(s) but also produce synergistic effects, as will be described in detail below.

## 3. Compound 968 Alone or in Combination Therapy

**Figure 3:**
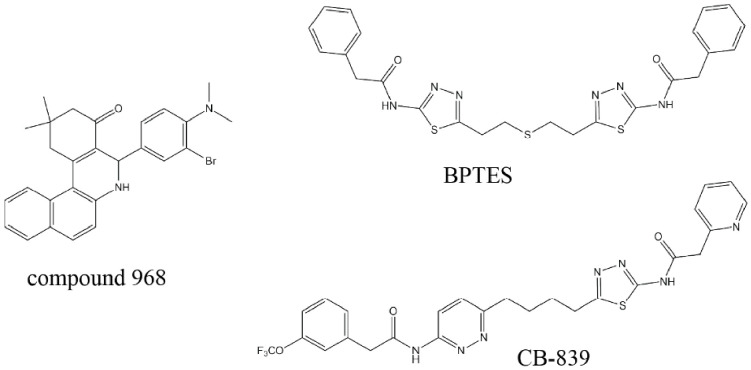
Non-competitive allosteric inhibitors of GA: compound 968 prevents the formation of active tetramers of GLS and GLS2. BPTES and CB-839 are isoform-specific and only bind to the interface between two dimers of the GLS tetramer. The three drugs block the conformational change required for catalytic turnover and GA activity.

Compound 968 is a dibenzophenanthridine, chemically 5-[3-bromo-4-(dimethylamino)phenyl]-2,3,5,6-tetrahydro-2,2-dimethyl-benzo[a]phenanthridin-4(1H)-one (Figure 3), which binds GLS and, unlike other inhibitors, also targets the GLS2 isoenzymes [21]. It is an allosteric modulator of GA, which induces apoptosis in cancer cells [36]. The pro-apoptotic effects of compound 968 have been demonstrated in both monotherapy and combination therapy settings [42]. For instance, the presence of compound 968 alone activates the release of the pro-apoptotic serine protease granzyme B, which is primarily secreted by activated T lymphocytes (CD3+, CD4+, and CD8+), boosting tumor infiltration and immune stimulation in ovarian cancer. This effect is further potentiated in vivo when combined with anti-PD-L1 antibodies, accompanied by an increase in the secretion of proinflammatory chemokines [42]. Positive laboratory results in immunotherapy do not always translate to the clinical setting. The reason for the poor effectiveness of some antibodies may be related to problems with their distribution and delivery to tumor cells due to biological and molecular barriers such as the extracellular matrix itself (made up mainly of collagens and glycoproteins), as well as the distinctive characteristics of the tumor microenvironment (TME) depending on the tumor type [43]. It is important to further optimize antitumor immunotherapy for ovarian carcinoma; while significant clinical results have been achieved in other cancer types—such as melanoma, colon cancer and non-small cell lung cancer (NSCLC)—therapeutic benefits have not been realized in others, including ovarian cancer [42]. In another study, the competitive mTORC allosteric inhibitor PP242 induced an increase in Gln metabolism. A combination of PP242 and GA inhibition by compound 968 in glioblastoma (GBM) patient-derived xenograft (PDX) models synergistically blocked tumor growth. Notably, no significant induction of cell death was observed in normal tissues, including the brain (cortex and hippocampus), liver, and kidney in mice treated with PP242, compound 968 alone, or the combination. These results demonstrate that GA inhibition can reverse mTORC-targeted therapy resistance in vivo by impairing tumor bioenergetics along with PP242 (Table 1), while maintaining low toxicity in normal tissues [44]. Of note, glutamate is a key metabolite affecting the TCA cycle and promoting resistance to mTOR inhibitor treatment, thereby enabling GBM cells survival. These findings may have important implications for combining mTORC kinase inhibitors with GA inhibition in patients with GBM and potentially other mTORC-activated cancers.

Another drug demonstrating greater efficacy in hepatic tumor cells than in normal ones, dihydroartemisinin—a semisynthetic derivative of artemisinin—has been shown to exert potent anticancer effects by increasing reactive oxygen species (ROS) levels (Table 1). Importantly, glutaminolysis and, therefore, GA activity, play a critical role in maintaining redox homeostasis, as GA-generated Glu serves for glutathione (GSH) synthesis both through direct incorporation and also by favoring cysteine (cystine) uptake via the xCT transporter (SLC7A11) as depicted in Figure 2. Cancer cells typically rely on glutaminolysis to boost antioxidant systems and support survival. Hence, the combination of dihydroartemisinin with compound 968 showed good efficacy to induce apoptosis, and synergistic capacity to decrease antioxidant power by disrupting redox homeostasis in cancer cells, while sparing normal cells [45]. The low required dose and low toxicity mean that this strategy meets the requirements for clinical translation. Similarly, inhibition of proliferation was selectively enhanced in cancer cells by compound 968, which inhibited tumor growth through down-regulation of the epidermal growth factor receptor (EGFR)/extracellular signal-regulated kinase (ERK) pathway and induction of G1/G0-phase cell cycle arrest [46]. The inhibitory effect on GA evoked the induction of autophagy by up-regulating beclin1 in NSCLC cells and was synergistically augmented when combined with chloroquine, protecting cells against apoptosis [46]. Interestingly, in the case of overcoming adriamycin chemoresistance, the effect appears not to be exclusively linked to GLS inhibition, since the same outcome is not achieved when compound 968 is replaced with another allosteric inhibitor, such as the drug CB-839, which specifically targets GLS isoforms [47]. The synergistic pro-apoptotic effect was potentiated by optimizing compound 968 through the synthesis of analogs that more effectively suppress P-glycoprotein, establishing a novel strategy to totally reverse multidrug resistance in breast cancer [47].

## 4. BPTES as an Effective GLS Inhibitor Against Cancer

BPTES is an allosteric inhibitor of GLS isoforms, chemically identified as bis-2-(5-phenylacetamido-1,2,4-thiadiazol-2-yl)ethyl (Figure 3). The efficacy of BPTES as a single antitumor agent has been demonstrated both in vitro and in vivo across several cancer types [48], although its highest potential is typically observed when used in combination with other drugs in multiple malignancies (Table 2). Tumor heterogeneity is a key factor in treatment design and optimization [35]. Therefore, understanding the gene expression profile of each specific tumor type is essential to decide which therapeutic strategy—or combination thereof—can achieve the greatest efficacy [32]. A study conducted at Columbia University provides a compelling example. Mutated NOTCH1, a common alteration in T-cell acute lymphoblastic leukemia (T-ALL), drives cell proliferation by regulating multiple pathways, including glutaminolysis. NOTCH1 signaling channels Gln metabolism into the tricarboxylic acid (TCA) cycle, promoting its use as a carbon source in NOTCH1-induced T-ALL. Dibenzozepine (DBZ), a gamma-secretase inhibitor (GSI), effectively blocks NOTCH1 activity. However, DBZ-treated leukemia cells that overexpress GLS show increased Gln utilization, suggesting that GLS overexpression may represent a potential mechanism of resistance to NOTCH1 inhibition. Combined treatment with DBZ and BPTES exhibited pronounced synergistic effects, impairing cell proliferation and sensitizing cells to NOTCH1 inhibition, with glutaminolysis assuming a dominant metabolic role over glycolysis. In xenograft mouse models, administration of BPTES plus DBZ resulted in strong tumor growth suppression. Notably, the Pten phenotype also appears to be a critical predictive factor for the efficacy of combined DBZ-BPTES therapy, as Pten-deleted T-ALL cells failed to response to GSI alone or in combination with GLS inhibition. Consequently, both GLS expression and Pten status could serve as predictive elements to guide treatment strategies in T-ALL [49]. The number of patients with NSCLC continues to rise year after year, while monotherapies fail to achieve the desired results—in part because only about 10% of patients exhibit the EGFR mutations that allow treatment with gefitinib. Combination therapy succeeds in inactivating CPSII through GA inhibition. This produces a synergistic effect that damages DNA and blocks pyrimidine synthesis via 5-FU in NSCLC [50]. Pyruvate dehydrogenase kinase (PDK) inhibition is a valuable strategy in some types of tumors, such as cervical cancer and colorectal cancer (CRC). For this purpose, one of the most widely used compounds is dichloroacetate, which also tends to be more effective in vivo than in vitro, probably due to the increased acidification of the TME. Characterization of the mechanism of action of dichloroacetate has shown that BPTES enhances antitumor activity in different cell models by inducing a glycolytic to oxidative shift, decreasing the PPP flux, and reactivating mitochondrial-dependent apoptosis in tumor cells that are dependent on mitochondrial oxidative phosphorylation (OXPHOS) for growth and proliferation [51]. TNBC represents the most aggressive histological subtype of breast cancer, has the poorest prognosis and is difficult to treat. Using cells models, dosages have been successfully optimized for each cell type by using sub-toxic concentrations of either etoposide or cisplatin in combination with BPTES [52], thereby opening a pathway that still requires optimization for clinical translation [43]. Another study has shown that N6-methyladenosine (m^6^A) readers play critical roles in the regulation of gene expression during TNBC progression. On the other hand, LRPPRC, a member of the pentatricopeptide repeat family, is an RNA-binding protein that targets lactate dehydrogenase A (LDHA) and regulates apoptosis, proliferation, metastasis and drug resistance in TNBC. The combination of LDHA inhibitor FX-11 and BPTES augmented the antiproliferative and invasive effect in both PDX and patient-derived organoids (PDO) models of TNBC [53]. Also in another TNBC model, BPTES pretreatment decreased GSH levels and enhanced loss of mitochondrial membrane potential, sensitizing tumor cells to oxidative damage and increasing apoptosis [54]. Similarly, in a pancreatic ductal adenocarcinoma (PDAC) model, a nanosystem was optimized to use ROS as an additional chemotherapeutic agent to improve the efficacy of BPTES and doxorubicin. These nanoparticles are released at the tumor site and have demonstrated therapeutic efficacy in both human organoid and mouse models [55]. Finally, inhibition of Gln metabolism by BPTES has proved an essential role in T-cell-mediated antitumor immunity. Thus, via a mechanism involving Fas/CD95 signaling, an increase in anti-PD-L1–mediated T-cell cytotoxicity was observed [56].

## 5. CB-839 Is the Most Successful GLS Inhibitor in Cancer Therapy

CB-839 (telaglenastat) is a derivative of BPTES, chemically 2-(pyridin-2-yl)-N-(5-(4-(6-(2-(3-(trifluoromethoxy)phenyl)acetamido)pyridazin-3-yl)butyl)-1,3,4-thiadiazol-2-yl)acetamide (Figure 3). CB-839 monotherapy has demonstrated significant antitumor activity both in vitro and in vivo [57,58]. However, it is important to note that dual or triple combination therapy has shown a synergistic increase in antiproliferative effects without causing greater damage to healthy cells, while also allowing dose reduction, for example with doxorubicin, which is associated with severe side effects in patients [59]. Although combination therapy is the preferred strategy in most antitumor approaches (Table 3), some studies report that a single nitrogen metabolism inhibitor, such as CB-839, can be even more effective than combining different inhibitors. For instance, in PDX of thyroid squamous cell carcinoma (SCC), CB-839 reduced the levels of the antioxidant enzyme NAD(P)H quinone oxidoreductase 1 (NQO1), thereby targeting and diminishing the master regulator nuclear factor erythroid 2-related factor 2 (NRF2) activity, which maintains redox homeostasis and protects cells from oxidative stress and damage. As an anticancer monotherapy, CB-839 was even more effective than the combination of the multireceptor tyrosine kinase inhibitor cabozantinib and the PI3K inhibitor GDC-0326 [60].

GLS inhibition by CB-839 leads to a decrease in mTOR activity and synergizes with mTOR inhibitors [39,61,62,63]. Interestingly, treatment of GLS-dependent TNBC cells with compound 968 did not decrease TCA cycle metabolite levels nor inhibit cancer cell proliferation, whereas CB-839 showed clear pharmacodynamic effects as well as tumor growth inhibition [61]. Similarly, combinations of CB-839 with everolimus or cabozantinib induced synergistic antiproliferative effects in vitro, and CB-839 enhanced the antiproliferative effects of everolimus, cabozantinib, sunitinib, or axitinib in vivo, supporting the targeting of glutaminolysis and glycolysis as a potent therapeutic strategy for renal cell carcinoma (RCC) [62]. On the other hand, tumor vascularization, which plays a crucial role in regulating the availability of glucose and Gln, should be evaluated to assess the efficacy of new metabolism-based therapies, particularly in resistant patient populations [63]. In addition, it is necessary to study alternative metabolic pathways that enable tumor cells to respond to metabolic stress and to inhibit these compensatory pathways in order to achieve synergistic clinical outcomes [64]. Accordingly, cancer cells undergo metabolic reprogramming of Gln metabolism to support tumor proliferation, and identifying vulnerabilities in this metabolic pathway can be used to develop novel strategies for cancer therapy, as described in NSCLC with the CDK inhibitor THZ1 [65]. Interestingly, a recent study found that CRC models developed resistance to CDK4/6 inhibitor palbociclib by upregulating glutaminolysis, and that combination with CB-839 overcame this resistance, producing synergistic effects [66]. Similar results were reported in lung adenocarcinoma models [67]. These studies highlight the relevance of Gln metabolism as a resistant and survival mechanism in cancer cells, due to its capacity to sustain multiple essential pathways. Therefore, GA inhibition appears to be a relevant adjuvant strategy, even in previously unsuspected contexts such as the CDK4/6 axis. In this case, although mechanisms are not yet fully elucidated, they likely involve downregulation of the E2F/Myc axis following CDK4/6 inhibition, thereby creating a synthetic lethal relationship with glutaminolysis and, consequently, with GA.

The combination of CB-839 with 5-FU inhibits cancer growth and overcomes resistance by activating NRF2 and increasing ROS levels, which trigger apoptosis through interconnected mechanisms involving BAX [68] and GPX4 [69], or by restoring energy homeostasis through the Gln pathway via activation of 5′ AMP-activated protein kinase (AMPK) [70]. Another drug that exhibits synergistic effect combined with CB-839 is cyclosporin A, which induces NRF2 ubiquitination and degradation [71]. These findings support the development of PROTACs (Proteolysis Targeting Chimeras), a revolutionary class of cancer therapeutics that, rather than simply inhibiting disease-causing proteins, selectively induce their degradation [63]. Furthermore, CB-839 plays a key role in overcoming resistance mechanisms in refractory liver cancer that arise with the iron chelator deferoxamine [72] and in pancreatic cancer following ATF4 silencing and ASCT2 inhibition [73]. Its antitumor activity was synergistically enhanced by the administration of chloroquine, which blocks autophagy and nucleotide metabolism in hepatic tumor cells [72] or with V-9302, which induced apoptosis in PDAC cells [73]. In a similar fashion, other drugs enhance chemosensitivity and therapeutic efficacy by blocking the uptake of key metabolites, thereby re-sensitizing cancer cells and overcoming multidrug resistance across different cancer types [74,75,76]. To further enhance cytotoxicity in combination therapies, an effective strategy is to modulate metabolic and oxidative stress using antioxidants, chemical agents, or radiotherapy, thereby potentiating the therapeutic effects of GA inhibition by telaglenastat [77,78,79,80] or bypassing the metabolic pathways that confer resistance in Gln-addicted cancers [81]. Furthermore, the successful treatment of resistant mutant tumors through targeting Gln metabolism also requires the identification of associated metabolic vulnerabilities, which may include epigenetic modifications [82]. T-cell-based and immune checkpoint inhibitor therapies are increasingly being combined with metabolic targeting strategies in refractory patients. The overexpression of inflammatory genes support GA plasticity between tumor cells and T cells and may generate an agonist response of CB-839 in T-cell-mediated antitumor responses [83,84]. As adaptive resistance commonly develops following treatment with therapeutic antibodies, it is essential to optimize the dose, regimen, and scheduling of combined or sequential therapies incorporating CB-839 to achieve maximum therapeutic efficacy [85]. Exploratory biomarker that determine responder with clinical benefit and one non-responder without clinical benefit will be equally essential to validate immunotherapeutic approaches that reinforce the integration of metabolic and immune-targeted approaches on Gln-dependent cancers [86]. Precision oncology studying the genetic and metabolic differences between individual resistant and sensitive tumors will be required for patients with refractory metastatic tumors [87]. Moreover, the development of robust biomarkers and in vivo exploratory techniques to assess metabolites profiles and drugs biodistribution will be indispensable for accurately evaluating the biological effects of these therapies in both tumor and normal tissues [88].

## 6. DON Returns Like a Trojan Horse

Although some recent studies have successfully used DON in combination therapy, for example, with MDiVi-1, a mitochondrial fission inhibitor that demonstrated antitumor efficacy in vitro and in vivo in ovarian cancer models [89], toxicity concerns have prevented DON from becoming the anti-Gln drug of choice. In 2016, a breakthrough opened a new avenue for DON use in the form of a prodrug, ethyl 2-(2-amino-4-methylpentanamido)-DON, named JHU083, with the critical advantage of inactive systemic administration of the compound and preferential accumulation and activation in the tumor microenvironment, allowing adequate concentrations at the site of action while minimizing side effects [20]. Since then, several studies have confirmed the utility of JHU083 in different tumor models (Table 4).

Mice treated with JHU083 alone or in combination with immunotherapy showed improved therapeutic outcomes, inducing a stronger antitumor response across several cancer types [90]. DON prodrug JHU083 monotherapy showed optimal biodistribution, even at brain level and significantly prolonged survival in mice bearing intracranial gliomas [91]. Combination therapy with radiation or other anticancer drugs may further extend survival, although additional studies are needed to fully determine the therapeutic potential of this Gln-targeting strategy in GBM. One of the major advantages of JHU083 is that it is preferentially activated within the TME, thereby increasing intratumoral drug exposure while reducing systemic toxicity. In addition, JHU083 promotes the infiltration of anti-tumor CD8^+^ or Th1 effector T cells into the tumor site [92]. Of note, the anticancer activity of JHU083 was based in the inhibition of the infiltration in both primary tumor and metastatic sites through a mechanism involving the reprogramming of tumor-associated macrophages (TAMs) from an immunosuppressive to a proinflammatory phenotype [93]. JHU083 treatment also increased CD8^+^ infiltration and promoted TAMs phenotype reprogramming in thyroid cancer. Therapeutic options for this malignancy remain limited, and DON has been shown to significantly inhibit tumor growth. However, because of its toxicity, the use of a prodrug such as JHU083 may represent a more suitable therapeutic approach. Indeed, JHU083, is substantially less toxic than DON and, at a dose of 2 mg/kg, demonstrated excellent antitumor efficacy in murine models [94]. Glioblastomas are also highly invasive and therapy-resistant. Novel combination therapies may provide promising strategies to overcome the limited efficacy of current chemotherapeutic approaches for this type of tumor. Among these, inhibition of GSH synthesis by L-buthionine sulfoximine (BSO) induced a compensatory metabolic reprogramming characterized by the upregulation of GLS. Therefore, the combination of BSO and JHU083 not only depleted GSH levels but also markedly impaired pyrimidine synthesis, resulting in both potent cytotoxic effect in GBM cells and tumor regression in vivo [95]. Adrenocortical carcinoma is another aggressive malignant neoplasm for which there are currently no effective medical treatments, and the overall survival of patients receiving cytotoxic chemotherapy is generally less than 15 months. However, nucleotide biosynthesis has also been identified as a metabolic vulnerability in this tumor, and JHU083 demonstrated excellent results when combined with an ATR kinase inhibitor [96]. Urological cancers also have limited therapeutic options, and immunotherapy has not yet achieved satisfactory clinical outcomes. Fortunately, the JHU083 monotherapy has successfully reprogrammed immunosuppressive TAMs and tumor-infiltrating monocytes in the TME toward a more pro-inflammatory phenotype, thereby suppressing tumor progression [97].

In 2019, another DON prodrug, (S)-isopropyl 2-((S)-2-acetamido-3-(1H-indol-3-yl)propanamido)-6-diazo-5-oxohexanoate, known as DRP-104 (sirpiglenastat), was developed. This prodrug retains the antitumor activity of DON while minimizing its systemic toxicity [98]. Notably, DRP-104 is bioactivated by serine proteases within the tumor, where it releases the active metabolite DON. In contrast, it is bioinactivated by carboxylesterases in healthy gastrointestinal tissues, generating a non-toxic molecule [24]. Table 5 summarizes the most representative studies demonstrating the antitumor activity of DRP-104 either as monotherapy or in combination with other therapeutic agents.

Treatment with DRP-104 alone or in combination with immune checkpoint inhibitors induced metabolic reprogramming of the TME by modulating cytokine production, altering TAMs phenotype and creating a more favorable immune milieu that enhanced both innate and adaptive immune responses [23]. PDAC is highly resistant to therapies due to its dense TME, which limits drug delivery, as well as its efficient DNA repair mechanisms and strong immunosuppressive properties [73]. As a compensatory response to the inhibition of Gln metabolism by DRP-104, which disrupts the synthesis of nucleotides, hexosamines and other amino acid-derived metabolites, the MAPK/ERK pathway is stimulated. Accordingly, the combination of DRP-104 with inhibitors targeting this pathway produced greater antitumor efficacy in vivo than either treatment alone [99]. Head and neck squamous cell carcinoma (HNSCC) is the sixth most common cancer worldwide, and dysregulated glutaminolysis is one of the key metabolic signatures distinguishing malignant from normal tissue [80]. DRP-104 impairs mTOR signaling, enhances autophagy, and induces lipid peroxidation, thereby sensitizing tumor cells to ROS. Therefore, its combination with RSL3, a ferroptosis inducer, not only enhanced the therapeutic vulnerabilities of HNSCC, but also synergistically attenuated tumor growth both in vitro and in vivo [100]. Tumors harboring KEAP1 mutations are particularly sensitive to Gln metabolism inhibition but frequently develop resistance to treatment, highlighting the need for combination therapies [60]. In lung cancer carrying this type of mutation, DRP-104 reduced tumor cell proliferation by impairing nucleotide biosynthesis while enhancing CD4 and CD8 T-cell function and improving the efficacy of immune checkpoint blockade. These effects translated into superior antitumor activity when DRP-104 was combined with anti-PD1 therapy [101]. In a prostate cancer model, DRP-104 monotherapy at minimally toxic doses (2 mg/kg/day) not only reduced cell proliferation, but also increased cell death through mechanisms involving both Gln-derived nitrogen and carbon metabolism [102]. In another prostate cancer model, adaptive resistance mechanisms enabled tumor cells to overcome the inhibition of purine biosynthesis. However, combining DRP-104 with a specific inhibitor of 5-methylthioadenosine phosphorylase targeted this metabolic vulnerability and resulted in superior therapeutic efficacy [103].

**Figure 4:**
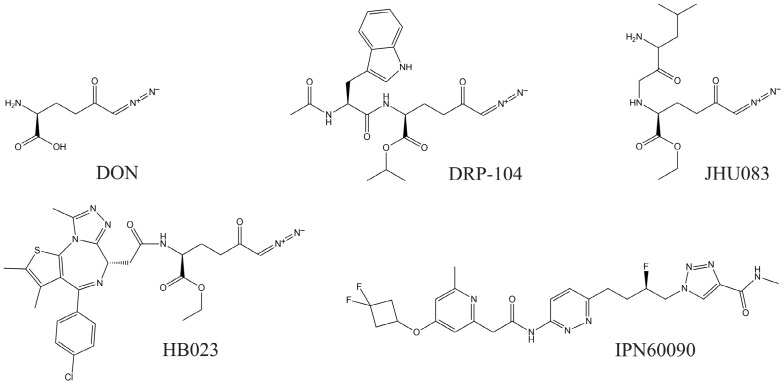
DON, prodrugs and new chemicals to block tumorigenesis: DON, DRP-104, JHU083, HB023, and IPN60090.

And, as if completing a circle, we return to the beginning with the chemical structure of DON (Figure 4). After decades of neglect, the inhibitory potential of DON has reemerged in the form of chimeric prodrugs that release the active compound—the Gln analog itself—directly at the tumor site to perform its precise function, while avoiding the drawbacks of its intrinsic toxicity. DON forms an irreversible covalent complex with GA by binding to the active-site serine residue and releasing the diazo group [104]. Thus, masked as precursors of the active agent, these prodrugs have once again highlighted the strong antitumor activity of DON. Interestingly, clinical trials are already underway for DRP-104 (NCT04471415, NCT06027086) and trials for JHU083 are also expected.

## 7. Targeting GA in Clinical Trials

As described above, the antitumor activity of CB-839 has been extensively characterized in multiple cell-based cancer models and in vivo (Section 5). Highly significant effects have been observed with both monotherapy [79,80] and combination therapy (Table 3). One of the most frequently reported antitumor effects is increased oxidative stress, often combined with radiotherapy for enhanced efficacy [79,80,105]. However, the options are very diverse, as demonstrated across various models confirming its value as an antitumor drug (Table 3). CB-839 has been the most studied GA inhibitor to date. Although its solubility and pharmacokinetic properties are suboptimal, its high specificity and potency have driven its inclusion in numerous clinical trials. Table 6 summarizes the most relevant clinical trials conducted to date. As many of these studies are still ongoing, primary and secondary outcome data remain unavailable in several cases; therefore, the key findings reported to date are presented. Notably, some clinical trials were terminated because of lack of efficacy. For example, trials evaluating nivolumab, a programmed cell death protein 1 (PD-1) inhibitor, in NSCLS and other advanced malignancies [105], as well as pembrolizumab in NSCLC [84]. As recently highlighted, several antibody-based therapies that demonstrated promising preclinical efficacy have failed to achieve comparable success in clinical oncology, primarily because of inadequate drug distribution within tumors [43]. Consequently, single-cell spatial pharmacobiology is expected to become increasingly valuable for patient stratification and biomarker-guided selection, enabling more robust comparisons between small-molecule and biologic strategies. This approach highlights therapeutic concepts with durable clinical relevance, minimal toxicity, high selectivity and reduced potential for adaptive escape [106]. These approaches will provide a more precise framework for assessing TME interactions in human tumors. Furthermore, they will enable the prediction of appropriate dosing regimens and a deeper understanding of resistance mechanisms specific to each individual tumor. No doubt, this paves the way for the near future development of single-cell spatial biomarkers in precision oncology.

Many articles highlight the need for a combined therapeutic approach that includes GA targeting, as Gln metabolism affects multiple cellular processes and metabolic pathways, including oxidative stress [113], DNA damage [114], response to radiotherapy [105], alterations in the harsh TME [115], and changes in the immune response [20,116]. A significant step toward clinical translation occurred in 2020 with the development of IPN60090 (or IACS-6274), a BPTES-derived molecule with the chemical structure 1-[(2R)-4-[6-[[2-[4-(3,3-difluorocyclobutyl)oxy-6-methylpyridin-2-yl]acetyl]amino]pyridazin-3-yl]-2-fluorobutyl]-N-methyltriazole-4-carboxamide. IPN60090 showed greater potency and selectivity than CB-839, with optimal pharmacokinetic properties characterized in mouse, rat, and dog [117]. This drug is currently being evaluated in a phase I clinical trial (NCT03894540). In epithelial ovarian cancer, IPN60090 has been shown to inhibit Zinc finger SWIM-type containing 4 (ZSWIM4), a transcription factor that induces drug resistance. Its effects have been characterized in vitro and in mouse xenografts, where it sensitized tumors to a CBP inhibitor (targeting CREB-binding protein and its paralog p300), and reduced tumor volume through an apoptosis/glutathione-dependent mechanism. These antitumor effects were synergistically augmented when combined with paclitaxel in PDO models [118]. In acute myeloid leukemia (AML) cells, IPN60090 induced apoptosis, inhibited tumor growth, and suppressed NADPH and ATP synthesis. Accordingly, it exhibited synergistic antiproliferative effects when combined with venetoclax, a BCL-2 inhibitor [78]. In a novel nanostrategy, IPN60090 was encapsulated in nanospheres targeting mitochondria, together with copper and IR780. While IPN60090 blocks GSH-mediated antioxidant defense, copper accentuates oxidative stress through cuproptosis, and IRP780 enhances the immune response and immunogenic cell death, achieving substantial synergistic antitumor effects in breast cancer cells both in vitro and in vivo [119].

## 8. Future Perspectives

Targeting Gln metabolism has been extensively validated as a powerful anticancer strategy [9,10,11,111,113,114,115,116]. In this context, targeting GA appears particularly promising, as it represents the main rate-limiting step of glutaminolysis, a pathway that provides bioenergetics, biosynthetic intermediates, and antioxidant protection. In fact, many cancers overexpress GA, particularly GLS, making its inhibition a prime opportunity to reverse cancer cell metabolic reprogramming, limit proliferation and render cells vulnerable to death induction, thereby enhancing sensitivity to chemoradiotherapy and improving therapeutic responses [10]. For immunotherapy-nonresponsive cancers, the prodrug JHU083 emerges as a promising novel treatment, even in the presence of immune-suppressing macrophages and scarce T-cells, positioning it as a potent option for tumors unresponsive to checkpoint inhibitors. In addition, it has demonstrated substantial therapeutic benefits in urological cancers, particularly by reducing angiogenesis in the TME [23].

Combinations aimed at boosting immune response are of particular interest here, since Gln depletion in the TME, caused by unrestrained incorporation and metabolism by Gln-addicted tumors, strongly limits lymphocyte activation and growth, which critically rely on Gln for clonal expansion, thereby constituting an immune escape mechanism for cancer cells [90,93]. Hence, limiting glutaminolysis by tumors increases Gln availability to immune cells in the TME, boosting immune response. Very recently, a new DON chimera has been designed by fusing DON with JQ1 to leverage JQ1’s well-established downregulation of PD-L1; chemically (6S)-4-(4-chlorophenyl)-2,3,9-trimethyl-6H-thieno[2-f]triazolo[3-a]diazepine-6-acetic acid 1,1-dimethylethyl ester, resulting in the new compound HB023 [120]. This new drug has shown great chemotherapeutic capacity in a murine model of colon cancer, overcoming tumor immune evasion and increasing anticancer efficacy by cooperatively activating both adaptive and innate antitumor immunity. Similar results have been observed in a murine model of clear cell renal cell carcinoma (ccRCC) with DRP-104, and with the GLS inhibitor CB-839 combined with PD-L1, achieving synergistic increases in antitumor immunity [121]. Indeed, anti-PD-1 and anti-PD-L1 therapies are revolutionizing cancer treatment, as drug resistance remains a major challenge; immune checkpoint inhibitors thus emerge as valuable tools to overcome multidrug resistance and collaborate with Gln metabolic reprogramming to achieve durable anticancer responses [23,42,44,83,84,90,93,94,101,116,121]. Collectively, the investigations described above emphasize the importance of combination therapy and the optimization of synergistic approaches, in which targeting Gln metabolism serves as a valuable tool.

Despite these promising results, the clinical translation of GLS inhibition remains limited by the remarkable metabolic plasticity of cancer cells and the emergence of multiple resistance mechanisms, including pathway activation, metabolic reprogramming, epigenetic and transcriptional adaptation, intratumoral heterogeneity and microenvironmental influences [106]. Tumors can adapt to reduced glutaminolysis by rewiring alternative metabolic pathways that preserve anabolic capacity and redox homeostasis, including increased utilization of alternative carbon sources, enhanced glucose-derived anaplerosis, or altered lipid metabolism [25,34]. Moreover, metabolic heterogeneity among tumors and even within the same tumor can strongly influence therapeutic response, as demonstrated in glioblastoma models showing reduced sensitivity to GLS inhibition due to metabolic adaptation [31,32]. Therefore, identifying metabolic dependencies before treatment will be essential for selecting patients most likely to benefit from GLS-targeted therapies. Genetic and molecular determinants also represent important regulators of resistance to Gln-targeted therapies [35]. The specific contribution of GA isoforms must be carefully considered, since GLS generally supports tumor growth, whereas GLS2 may exert context-dependent tumor-suppressive functions [27,28,113]. Consequently, isoenzyme-specific inhibition may be required to maximize therapeutic efficacy while minimizing undesirable effects. In addition, oncogenic alterations such as KRAS mutations can reshape Gln metabolism and influence tumor dependency on this nutrient, potentially affecting responses to GLS-targeted therapies [31,32,58]. Similarly, genetic alterations such as STK11/Lkb1 loss and KEAP1 mutations can modulate the response to Gln-targeted therapies in KRAS-driven lung cancer, highlighting the complex interplay between tumor-intrinsic metabolism and antitumor immunity [122]. Notably, Gln metabolism is also required for CD8+ T-cell activation and effector function, suggesting that GLS inhibition may have context-dependent effects on immune responses [101]. These observations highlight the need to integrate genomic, metabolic, and immunological biomarkers when designing personalized strategies targeting Gln metabolism. Another important challenge is the identification of mechanisms regulating resistance within the TME. Although Gln blockade can reduce tumor-mediated metabolic suppression and enhance immune responses, immune cells also require Gln metabolism for proliferation, activation and effector functions [90,93,122]. Therefore, excessive or non-selective inhibition may compromise antitumor immunity depending on tumor type, immune composition, dose, and treatment combination. Future approaches should aim to preferentially disrupt tumor Gln dependence while preserving immune cell functionality, potentially through tumor-targeted delivery systems, prodrug formulations, or rational combinations with immunotherapies [23,24].

To overcome this challenge, metabolic imaging approaches may provide valuable tools for patient stratification. Notably, Gln-based positron emission tomography (Gln-PET) imaging using (2S, 4R)-4-[^18^F]Gln has proven effective for tumor diagnosis [123], disease tracking [124], therapeutic monitoring [125], and pharmacodynamic of drugs such as CB-839 [89]. This technique exhibits the highest reproducibility and repeatability in preclinical cancer models [126], and has been validated in a phase I/II clinical trial of CB-839 (NCT03263429) in patients with metastatic CRC [86]. In personalized oncology, Gln-PET enables organ- and patient-specific monitoring based on the organ being studied and the patient [88], including analysis of the TME profiling, as demonstrated in a mouse model and a patient with brain metastases from invasive ductal breast carcinoma [127]. In addition to addressing some of the main challenges in antitumor therapy, such as cancer heterogeneity, drug resistance, and immune evasion, nano-delivery strategies will be essential for modulating the TME through combination therapy and immunometabolic crosstalk [128]. Of particular interest are chimeric compounds that exhibit immunometabolic bifunctionality. For instance, Zhang et al. combined the prodrug JHU083 (which blocks Gln metabolism) with MSA-2, an agonist of the interferon gene activation pathway that regulates innate immunity in the TME and elicits a robust immunogenic response [129]. This bifunctional construct has demonstrated very promising results in vitro and in vivo in colon cancer models, with synergistic enhancement when combined with 5-FU or anti-PD-L1 therapy [129]. Nevertheless, future research must study how, for every single cancer, Gln blockage can support or impair antitumor immunity, depending on tumor type, immune context, dosage, compartment and treatment combination.

## 9. Conclusions

Many tumors exhibit significant intrinsic plasticity, triggering metabolic reprogramming to develop drug resistance and enhance biosynthetic and signaling pathways that sustain their proliferation program [130,131]. Therefore, combination therapy represents one of the few viable strategies for successful neoplasm treatment (Table 1, Table 2, Table 3, Table 4 and Table 5). Several reviews have described the activity of multiple GA inhibitors [9,10,11,111], as Gln metabolism constitutes a central network for tumor growth and survival, providing energy and essential substrates [34,116,132]. In this work we emphasize the need to design targeted therapeutic interventions based on blocking and/or activating different metabolic and/or cell signaling pathways looking to counteract the great adaptability of tumors to oxidative and/or metabolic stress conditions. Inhibition of GA, either through allosteric inhibitors (i.e.,: compound 968, BPTES, CB-839, IPN60090) or Gln analogs (i.e.,: DON, JHU083, DRP-104, HB023) represents a useful intervention targeting glutaminolysis, which as discussed constitutes an essential pathway for cancer survival and progression. This approach offers dual advantages: synergistic efficacy with a broad range of drugs, and reduced dosing requirements for certain agents, thereby minimizing toxicity to healthy cells. Combination therapy can integrate Gln metabolism inhibitors with other chemotherapeutic drugs, radiotherapy, or immunotherapy, optimizing overall efficacy by targeting multiple pathways while mitigating resistance development [133]. Recent research and ongoing clinical trials position CB-839 and DRP-104 as promising drugs targeting nitrogen metabolism, either by blocking GA (CB-839) or inhibiting Gln across multiple enzymes (DRP-104) [34]. Both demonstrate efficacy as monotherapy but excel in combination regimens, with CB-839 notably restricting TCA cycle flux, and DRP-104 broadly suppressing proliferative nodes like purine biosynthesis [134]. Moreover, DRP-104 uniquely modulates antitumor immune activity [24] by reshaping the TME [23,101]. A dysregulated, tumor-supportive TME represents a hallmark of cancer [135], and targeting Gln metabolism shows great potential for abrogating the tumor-supportive environment in both preclinical models [89] and patients [127]. Efforts to optimize inhibitors—including prodrug formulations—continue, exemplified by a tert-butyl ester derivative of DRP-104 that offers improved solubility and in vivo stability [136].

The above findings underscore the value of identifying possible targeted therapy-induced resistance mechanisms to design novel combination approaches that achieves the previously discussed advantages. Recent strategies have proposed integrating Gln metabolism into cancer diagnosis, classification, treatment, and monitoring [25]. In animal models, many tumors are especially dependent on Gln metabolism [32]. This observation suggests that imaging, quantifying, or blocking Gln metabolism in human cancers could be incorporated into the diagnosis and management of the disease [137], including PET-based technologies [89,121,138]. In most cases, it would be beneficial to use in vivo perioperative administration of isotope-labeled biomarkers (glucose and Gln) to cancer patients to differentiate metabolic pathways between tumors and benign tissue [139]. Additionally, in vivo Gln metabolic studies may help predict which tumors will respond to metabolism-targeted therapies [2].

**Figure 5:**
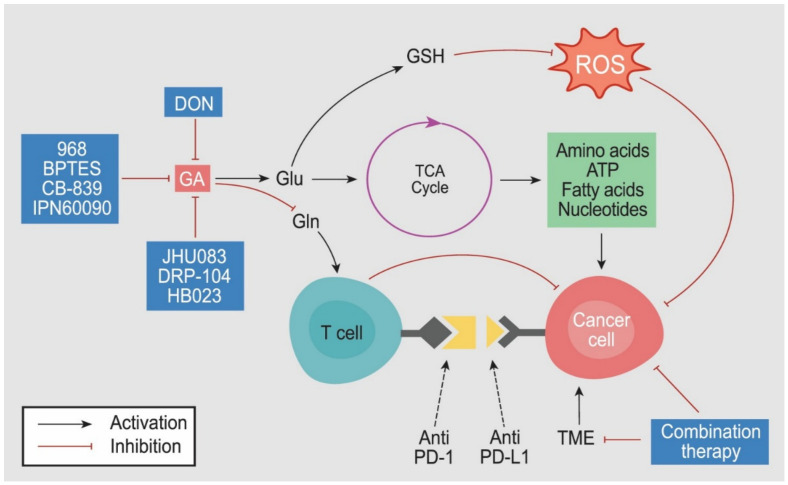
Mechanisms targeting cancer cells in combination therapy, together with ROS enhancement and immunotherapy. Blocking GA constitutes an essential component of the combination therapy, which may also incorporate immunotherapeutic drugs, such as checkpoint inhibitors to prevent PD-1/PD-L1 interaction, thereby enabling the immune system to recognize and attack tumor cells. Additional and synergistic effects can be achieved by targeting metabolic reprogramming that resets the tumor microenvironment for cancer growth. In this context, blocking GA allows for greater availability of Gln in the TME, an essential nutrient for T-cells. TCA cycle is depicted in purple. Dashed arrows indicate respective antibodies. ATP, adenosine triphosphate. GA, glutaminase. Gln, glutamine. Glu, glutamate. GSH, glutathione. PD-1, programmed cell death protein 1. PD-L1, programmed cell death ligand 1. ROS, reactive oxygen species. TCA, tricarboxylic acid. TME, tumor microenvironment.

In contrast, it should not be overlooked that several studies have identified important limitations and challenges associated with glutaminase inhibitors, including: (i) the need for isoenzyme-specific GLS inhibition when GLS2 acts as a tumor suppressor [28]; (ii) intestinal side effects caused by mucosal damage and cell death following GA inhibition [22]; (iii) metabolic reprogramming in some tumors through increased lipid oxidation to compensate for reduced glutaminolysis, thereby limiting the efficacy of GA inhibition [34]; (iv) compensatory increases in glucose metabolism through pyruvate carboxylase [25]; (v) the ability of some KRAS-mutant cancers to acquire Gln through alternative mechanisms, reducing the effectiveness of GA as a therapeutic target [86,133]; (vi) the incompatibility of GA inhibition with KEAP1 and STK11/Lkb1 co-mutations in KRAS-mutant lung adenocarcinoma because glutamate is required for CD8 T-cell activation [122]; and (vii) the high dependence of immune-system T cells on Gln metabolism for proliferation, requiring careful evaluation of GA blockade to achieve a balanced therapeutic response [140]. Hence, further research is needed to elucidate the metabolic consequences of GLS inhibition in combination with immunotherapy, as well as potential resistance mechanisms and side effects. However, there are also highly promising therapeutic findings, for example in ovarian cancer [42] as well as in lung and colon cancer [56]. For possible personalized use in the future a strong and in-depth study must be conducted, including tumor type, selected biomarkers, immune context, glutamine dependency, GLS versus GLS2 biology, metabolic plasticity, resistance mechanisms, and adequate patient selection. In addition, understanding the genetic and epigenetic circuits regulating GLS will facilitate the development of improved combination therapies that exploits Gln metabolism reprogramming for immune evasion, ultimately leading to novel anticancer drugs and precision oncology approaches [90,116,130,141,142]. In this field, the most promising research strategies appear to integrate anti-PD-1/PD-L1 therapies with targeting of metabolic interactions within the TME that promote tumorigenesis (Figure 5) [143]. Indeed, several very recent analyses have identified compelling combination therapy approaches showing clinical efficacy [144].

## Figures and Tables

**Table 1:** Relevant drug combination studies involving the GA inhibitor compound 968.

Drug	Model	Key Effect(s) ^1^	Reference
PP242	GBM ^2^ mice xenografts	Inhibiting mTORC1 ^3^	[44]
Dihydroartemisinin	HCC ^4^ in vitro	Activating apoptosis by increasing ROS ^5^	[45]
Chloroquine	NSCL5 ^6^ in vitro	Inhibiting autophagy	[46]
Adriamycin	MCF-7 breast cancer cells in vitro	Inhibiting P-gp ^7^ and overcoming drug resistance	[47]
Anti-PD-L1 ^8^	Ovarian cancer in vitro and in mice xenografts	Increasing apoptosis and immune response	[42]

^1^ Key effect(s) in addition to glutaminase inhibition are remarked. ^2^ GBM, glioblastoma multiforme. ^3^ mTORC1, mammalian target of rapamycin complex 1. ^4^ HCC, hepatocellular carcinoma. ^5^ ROS, reactive oxygen species. ^6^ NSCLC, non-small cell lung cancer. ^7^ P-gp, P-glycoprotein. ^8^ PD-L1, programmed cell death ligand 1.

**Table 2:** Chemicals used in combination with the GLS inhibitor BPTES that exhibit promising effects in antitumor therapy.

Drug(s)/Agent(s)	Model(s)	Key Effect(s) ^1^	Reference
Dibenzozepine	ALL ^2^ mice xenografts	γ-secretase inhibition, NOTCH1 ^3^ cleavage, and activation of autophagy	[49]
5-Fluorouracil	NSCLC ^4^ in vitro	Inhibiting thymidylate synthase and CPSII ^5^	[50]
Dichloroacetate	CRC ^7^ and cervical cancer cells in vitro	Decrease in PPP ^8^ and activation of apoptosis	[51]
Etoposide and cisplatin	TNBC ^6^ in vitro	Activating apoptosis by a BAX/BCL-2 mechanism	[52]
Bicalutamide	Prostate cancer in vitro and in rat xenografts	Blocking AR ^9^ and lipid metabolism	[48]
FX-11	TNBC ^6^ mice xenografts and PDO ^10^	Inhibiting LDHA ^11^	[53]
PAM ^12^ (ROS ^13^)	TNBC ^6^ in vitro	Activating apoptosis by DNA damage and inhibiting ATP ^14^ production	[54]
Doxorubicin, Fe^3+^, EGCG ^15^ and BPTES nanoparticles	PDAC ^16^ in vitro, mice xenografts, and PDO ^9^	Activating apoptosis by increasing ROS ^12^ and DNA damage	[55]
Anti-PD-L1 ^17^	Lung and colon cancer in vitro and in mice xenografts	Increasing Fas expression showing a synergistic antitumor effect	[56]

^1^ Key effect(s) in addition to GLS inhibition are indicated. ^2^ ALL, acute lymphoblastic leukemia. ^3^ NOTCH1, neurogenic locus notch homolog protein 1. ^4^ HNSCC, head and neck squamous cell carcinoma. ^5^ CPSII, carbamoyl phosphate synthetase II. ^6^ TNBC, triple-negative breast cancer. ^7^ CRC, colorectal cancer. ^8^ PPP, pentose phosphate pathway. ^9^ AR, androgen receptor. ^10^ PDO, patient-derived organoids. ^11^ LDHA, lactate dehydrogenase A. ^12^ PAM, plasma-activated medium. ^13^ ROS, reactive oxygen species. ^14^ ATP, adenosine triphosphate. ^15^ EGCG, epigallocatechin gallate. ^16^ PDAC, pancreatic ductal adenocarcinoma. ^17^ PD-L1, programmed cell death ligand 1.

**Table 3:** Studies demonstrating promising efficacy of GLS inhibitor CB-839 in combination therapy.

Drug(s)	Model(s)	Key Effect(s) ^1^	Reference
AZD8055	TNBC ^2^ mice xenografts	Inhibiting mTORC1 ^3^	[61]
Everolimus	RCC ^4^ mice xenografts	Inhibiting mTORC1 ^3^	[62]
MLN128	Mice xenografts of lung SCC ^5^, HNSCC ^6^ and osteosarcoma	Inhibiting mTORC1 ^3^	[39]
TAK228	Lung SCC ^5^ mice xenografts	Inhibiting mTORC1 ^3^	[63]
CPI-613	2D culture, 3D culture, and mice xenografts of HNSCC ^6^	Inhibiting TCA ^7^ cycle	[64]
THZ1	NSCLC ^8^ in vitro	Inhibiting CDK7 ^9^	[65]
Palbociclib	CRC ^10^ mice xenografts	Inhibiting CDK4/6 ^11^	[66]
Palbociclib	Lung adenocarcinoma cells	Inhibiting CDK4/6 ^11^	[67]
5-Fluorouracil	CRC ^10^ mice xenografts	Inhibiting thymidylate synthase and inducing IL-8 ^12^ to attract neutrophils into the tumor	[68]
5-Fluorouracil	HCC ^13^ mice xenografts	Inhibiting thymidylate synthase, enhancing oxidative stress, and increasing ferroptosis	[69]
5-Fluorouracil and cisplatin	ESCC ^14^ in vitro and mice xenografts	Increasing apoptosis targeting TIGAR ^15^	[70]
Cyclosporin A	NSCLC ^8^ mice xenografts	Inducing NRF2 ^16^	[71]
Deferoxamine	HCC ^13^ in vitro	Iron deficiency	[72]
V-9302	PDAC ^17^ in vitro	Inhibiting ASCT2 ^18^ Gln transport	[73]
DRB-18	ICC ^19^ in vitro	Inhibiting glucose transport	[74]
Ursodeoxycholic acid	Liposarcoma mice xenografts	Inhibiting SLC7A11 ^20^ cystine transport and GSH synthesis	[75]
Aspirin	CRC ^10^ mice xenografts	Inhibiting SLC7A11 ^20^ and SLC7A5 ^21^	[76]
EGCG ^22^	Multiple myeloma in vitro	Activating apoptosis by a BAX/BCL-2 mechanism	[77]
Venetoclax	AML^23^ in vitro	Inhibiting BCL-2	[78]
BSO ^24^, auranofin, RT ^25^	Cervix cancer in vitro and mice xenografts	Increasing oxidative stress	[79]
RT ^25^	HNSCC ^6^ in vitro and mice xenografts	Increasing oxidative DNA damage and apoptosis	[80]
Dihydroartemisinin	GBM ^26^ in vitro	Increasing oxidative stress and apoptosis	[33]
Oxamate, D609, doxorubicin	Breast cancer in vitro	Inhibiting LDH ^27^ and PC-PLC ^28^	[59]
ENZA ^29^, IACS ^30^	Prostate cancer cells in vitro and blood cells from patients with prostate cancer	Increasing ROS ^31^ and decreasing oxidative phosphorylation	[57]
V-9202	HCC ^13^ in vitro and mice xenografts	Lowering Gln transport and GSH ^32^, increasing ROS ^31^, and apoptosis	[81]
Selumetinib	NSCLC ^8^ in vitro and mice xenografts	Inhibiting ERK ^33^, increasing ROS ^31^ and autophagy	[58]
Osimertinib	Lung adenocarcinoma in vitro	Inhibiting tyrosine kinase	[82]
Sunitinib or axitinib	RCC ^4^ mice xenografts	Inhibiting tyrosine kinase	[62]
anti-PD-1 ^34^ or anti-PD-L1 ^35^	Mice bearing syngeneic colon carcinoma	Inhibiting immune checkpoint proteins and ligands	[83]
anti-CD152 ^36^ or anti-PD-1 ^34^	Melanoma cells in vitro and mice xenografts	Activating T-cell-mediated immunotherapy	[84]
Bevacizumab	Ovarian cancer mice xenografts	Inhibiting VEGF ^37^	[85]
Cabozantinib	RCC ^3^ mice xenografts	Inhibiting VEGFR ^38^	[62]
Panitumumab	Metastatic RCC ^3^ patients	Inhibiting EGFR ^39^	[86]
Cetuximab	CRC ^10^ in vitro and mice xenografts	Inhibiting EGFR ^39^	[87]
Metformin	Osteosarcoma in vitro and in mice xenografts	Disrupting metabolism	[88]

^1^ Key effect(s) in addition to glutaminase inhibition are indicated. ^2^ TNBC, triple-negative breast cancer. ^3^ mTORC1, mammalian target of rapamycin complex 1.^4^ RCC, renal cell carcinoma. ^5^ SCCC, squamous cell carcinoma. ^6^ HNSCC, head and neck squamous cell carcinoma. ^7^ TCA, tricarboxylic acid. ^8^ NSCLC, non-small cell lung cancer. ^9^ CDK7, cyclin-dependent kinase 7. ^10^ CRC, colorectal cancer. ^11^ CDK4/6, cyclin-dependent kinase 4/6. ^12^ IL-8, interleukin-8. ^13^ HCC, hepatocellular carcinoma. ^14^ ESCC, esophageal squamous cell carcinoma. ^15^ TIGAR, TP53-induced glycolysis and apoptosis regulator. ^16^ NRF2, nuclear factor erythroid 2-related factor 2. ^17^ PDAC, human pancreatic adenocarcinoma. ^18^ ASCT2, Alanine Serine Cysteine Transporter 2. ^19^ ICC, intrahepatic cholangiocarcinoma. ^20^ SLC7A11, Solute Carrier Family 7 Member 11. ^21^ SLC7A5, Solute Carrier Family 7 Member 5. ^22^ EGCG, epigallocatechin gallate. ^23^ AML, acute myeloid leukemia. ^24^ BSO, L-buthionine sulfoximine. ^25^ RT, radiation. ^26^ GBM, glioblastoma multiforme. ^27^ LDH, lactate dehydrogenase, ^28^ PC-PLC, phosphatidylcholine-specific phospholipase C. ^29^ ENZA, anti-androgen enzalutamide. ^30^ IACS, mitochondrial electron transport component complex I inhibitor IACS-010759. ^31^ ROS, reactive oxygen species. ^32^ GSH, glutathione. ^33^ ERK, extracellular signal-regulated kinase. ^34^ PD-1, programmed cell death protein 1. ^35^ PD-L1, programmed cell death ligand 1. ^36^ CD152, cluster of differentiation 152. ^37^ VEGF, vascular endothelial growth factor. ^38^ VEGFR, vascular endothelial growth factor receptor. ^39^ EGFR, epidermal growth factor receptor.

**Table 4:** JHU083 as a novel DON prodrug for antitumor therapy.

Additional Drug	Model	Key Effect(s) ^1^	Reference
Anti-PD-1 ^2^	Mice models bearing colon cancer, lymphoma, and melanoma	Increasing apoptosis and inhibiting antitumor immune response	[90]
None	GBM ^3^ in vitro and in orthotopic mice	Inhibiting mTORC1 ^4^	[91]
EVax ^5^	Lung transgenic mice	Inhibiting EGFR ^6^	[92]
Anti-CD152 ^7^ or anti-PD-1 ^2^	Myeloid cells and mice xenografts	Activating T-cell-mediated immunotherapy	[93]
None	Thyroid cancer mice xenografts	Inhibiting CD47 ^8^ and PD-L1 ^9^	[94]
BSO ^10^	GBM ^3^ in vitro and mice intracranial xenografts	Inhibiting GSH ^11^-dependent antioxidant capacity	[95]
Elimusertib	ACC ^12^ in vitro and mice xenografts	Inhibiting DNA damage response	[96]
None	Prostate and bladder cancer in vitro and syngeneic heterotopic mouse models	Increasing apoptosis and decreasing TCA ^13^ cycle and purine metabolism	[97]

^1^ Key effect(s) in addition to glutamine metabolism inhibition is remarked. ^2^ PD-1, programmed cell death protein. ^3^ GBM, glioblastoma multiforme. ^4^ mTORC1, mammalian target of rapamycin complex 1. ^5^ EVax, EGFR^6^ peptide vaccine. ^6^ EGFR, epidermal growth factor receptor. ^7^ CD152, cluster of differentiation 152. ^8^ CD47, cluster of differentiation 47. ^9^ PD-L1, programmed cell death ligand 1. ^10^ BSO, buthionine sulfoximine. ^11^ GSH, glutathione. ^12^ Adrenocortical Carcinoma. ^13^ TCA, tricarboxylic acid.

**Table 5:** DRP-104 as a novel DON prodrug for antitumor therapy.

Additional Drug	Model	Key Effect(s) ^1^	Reference
Anti-PD-1 ^2^	CADC ^3^ in vitro and mice xenografts	Modulation of the TME ^4^	[23]
Trametinib	PDAC ^5^ in vitro and syngeneic mice model	Inhibiting MAPK ^6^ and ERK ^7^ kinase 1/2	[99]
RSL3 ^8^	HNSCC ^9^ in vitro and mice xenografts	Inhibition of GPX4 ^10^ and activation of ferroptosis	[100]
Anti-PD-1 ^2^	Orthotopic lung cancer mice	Inhibiting cytokines and increasing antitumor T cell response	[101]
None	Prostate cancer in vitro and mice xenografts	Activation of apoptosis, targeting TCA ^11^ cycle and nucleotide synthesis	[102]
MTDIA ^12^	Prostate cancer in vitro and mice xenografts	Inhibition of MTAP ^13^	[103]

^1^ Key effect(s) in addition to glutamine metabolism inhibition is remarked. ^2^ PD-1, programmed cell death antibody. ^3^ CADC, colon adenocarcinoma. ^4^ TME, tumor microenvironment. ^5^ PDAC, pancreatic ductal adenocarcinoma. ^6^ MAPK, mitogen-activated protein kinase. ^7^ ERK, extracellular signal-regulated kinase. ^8^ RSL3, RAS-selective letal 3. ^9^ HNSCC, head and neck squamous cell carcinoma. ^10^ GPX4, glutathione peroxidase 4. ^11^ TCA, tricarboxylic acid. ^12^ MTDIA, inhibitor of MTAP ^13^. ^13^ MTAP, methylthioadenosine phosphorylase.

**Table 6:** Key clinical trials evaluating telaglenastat (CB-839), a glutaminase inhibitor, in combination therapies for cancer treatment.

Identifier	Phase	Cancer Type	No. Patients	Dose and Schedule	Combination Regimen	Main Outcomes	Reference
NCT03872427	II	Solid tumors or metastatic/unresectable malignant peripheral nerve sheath tumors	54	800 mg, BID ^1^	Monotherapy	Treatment was well tolerated	[107]
NCT03263429	II	Metastatic and refractory RAS CRC ^2^	29	400–600–800 mg, BID ^1^	Panitumumab (6 mg/kg, QD ^3^), irinotecan (180 mg/m^2^, QD ^3^)	Triplet regimen well tolerated	[87,107]
NCT03047993	II	Advanced myelodysplastic syndrome	28	600 mg, BID ^1^	Azacitidine (75 mg/m^2^, 7 days/cycle)	Response rate 70%; complete remission rate 53%	[107,108]
NCT02861300	II	Metastatic PIK3CA ^4^ mutant CRC ^2^	50	800 mg, BID ^1^	Capecitabine (750–1000 mg/m^2^, QD ^3^)	Well tolerated at biologically active doses	[107]
NCT03057600	II	Advanced TNBC ^5^	52	800 mg, BID ^1^	Paclitaxel (175 mg/m^2^, 3 times/cycle)	Trial discontinued because of disease progression	[109]
NCT03428217	II	Advanced or metastatic RCC ^6^	444	800 mg, QD ^3^	Cabozantinib (60 mg, 1 time/cycle)	At the time of termination, 182 deaths had occurred	[62,109]
NCT03965845	II	Advanced or metastatic CRC ^2^ and NSCLC ^7^	53	400–600–800 mg, BID ^1^	Palbociclib (75–125 mg, days 1–21 of cycle)	Results have not yet been submitted	[66,110]
NCT03831932	II	Metastatic and EGFR ^8^ activating mutation NSCLC ^7^	22	800 mg, BID ^1^	Osimertinib (80 mg, days 1 and 16)	Acceptable safety profile in both mono and combination therapy	[82,110]
NCT03163667	II	Metastatic ccRCC ^9^	69	800 mg, BID ^1^	Everolimus (5–10 mg, QD ^3^)	PFS ^10^ was approximately doubled compared with placebo	[62,110]
NCT04250545	I	Advanced and metastatic NSCLC ^7^	22	400–600–800 mg, BID ^1^	Sapanisertib (2–3 mg, 1 time/cycle)	PFS ^10^ approximately doubled compared with placebo, but serious adverse events reported	[110]
NCT03798678	I	Recurrent or refractory multiple myeloma	36	400–600–800 mg, BID ^1^	Carfilzomib (30 mg, 5 times/cycle), dexamethasone (1.5 mg, 7 times/cycle)	Encouraging clinical activity	[111]
NCT03528642	I	IDH ^11^-mutated diffuse or anaplastic astrocytoma	40	800 mg, BID ^1^	Temozolomide (75 mg/m^2^, QD ^3^); radiation (2 Gy QD ^3^)	Improved efficacy compared with standard therapy	[111]
NCT05521997	II	Advanced cervical cancer	42	800 mg, BID ^1^	Cisplatin (20 mg/m^2^, 1 day/week), radiation (2 Gy, 4 days/week)	Not yet recruiting	[105]
NCT02771626	II	Advanced melanoma, ccRCC ^9^ and NSCLC ^7^	118	600–800 mg, BID ^1^	Nivolumab (240–360 mg intravenous every 2 weeks)	Non-serious adverse events. Lack of clinical benefit	[109]
NCT04265534	II	Metastatic KEAP1 ^12^/NRF2 ^13^-mutated, nonsquamous NSCLC ^7^	40	800 mg, BID ^1^	Pembrolizumab (165 mg, intravenous every 3 weeks)	Non-serious adverse events. Lack of clinical benefit	[84,112]

^1^ BID, twice daily. ^2^ CRC, colorectal cancer. ^3^ QD, one daily. ^4^ PIK3CA, phosphatidylinositol-4,5-bisphosphate 3-kinase catalytic subunit alpha. ^5^ TNBC, triple-negative breast cancer. ^6^ RCC, renal cell carcinoma. ^7^ NSCLC, non-small cell lung cancer. ^8^ EGFR, epidermal growth factor receptor. ^9^ ccRCC, clear cell renal cell carcinoma. ^10^ PFS, progression free survival. ^11^ IDH, isocitrate dehydrogenase. ^12^ KEAP1, Kelch-like ECH-associated protein 1. ^13^ NRF2, nuclear factor erythroid 2-related factor 2.

## Data Availability

All data generated in this study are available from the corresponding authors upon request.

## Abbreviations

5-FU	5-fluorouracil
ALL	Acute lymphoblastic leukemia
AML	Acute myeloid leukemia
AMPK	5′ AMP-activated protein kinase
ATP	Adenosine triphosphate
BSO	L-buthionine sulfoximine
ccRCC	Clear cell renal cell carcinoma
CRC	Colorectal cancer
DBZ	Dibenzozepine
EGFR	Epidermal growth factor receptor
ERK	Extracellular signal-regulated kinase
GA	Glutaminase
GBM	Glioblastoma
Gln	Glutamine
Glu	Glutamate
GLS	Glutaminase isoenzyme 1
GLS2	Glutaminase isoenzyme 2
GS	Glutamine synthetase
GSH	Glutathione
GSI	Gamma-secretase inhibitor
HNSCC	Head and neck squamous cell carcinomas
LDHA	Lactate dehydrogenase A
NQO1	NAD(P)H quinone oxidoreductase 1
NRF2	Nuclear factor erythroid 2-related factor 2
NSCLC	Non-small cell lung cancer
OXPHOS	Oxidative phosphorylation
PD-1	Programmed cell death protein 1
PET	Positron emission tomography
PDK	Pyruvate dehydrogenase kinase
PDAC	Pancreatic ductal adenocarcinoma
PDO	Patient-derived organoids
PDX	Patient-derived xenografts
PD-1	Programmed cell death protein 1
PD-L1	Programmed cell death ligand 1
PROTAC	Proteolysis Targeting Chimera
RCC	Renal cell carcinoma
ROS	Reactive oxygen species
SCC	Squamous cell carcinoma
T-ALL	T-cell acute lymphoblastic leukemia
TAM	Tumor-associated macrophage
TCA	Tricarboxylic acid
TKI	Tyrosine kinase inhibitor
TME	Tumor microenvironment
TNBC	Triple negative breast cancer
